# Aggressive Posterior Retinopathy of Prematurity (APROP): LASER as the Primary Modality of Treatment

**DOI:** 10.18502/jovr.v16i3.9437

**Published:** 2021-07-29

**Authors:** Shilpi H Narnaware, Prashant K Bawankule, Dhananjay Raje

**Affiliations:** ^1^Consultant Vitreo-retina & ROP Specialist, Sarakshi Netralaya, Rajiv Nagar, Wardha Road, Nagpur 440025, Maharashtra, India; ^2^Vitreo-Retinal Surgeon, Rajiv Nagar, Wardha Road, Nagpur 440025 Maharashtra, India; ^3^Head, Data Analysis Group, MDS Bio-analytics Pvt. Ltd., Shankar Nagar, Nagpur 440010, Maharashtra, India

**Keywords:** Aggressive Posterior Retinopathy of Prematurity, LASERS, Success Rate

## Abstract

**Purpose:**

To study the success rate of LASER as a primary modality of treatment in aggressive posterior retinopathy of prematurity (APROP) cases.

**Methods:**

This is a prospective case series of 56 eyes of 28 preterm babies (males = 21) with APROP who underwent laser therapy. Babies were divided into groups on the basis of gestational age (GA), birth weight (BW), and postmenstrual age (PMA) at which treatment was performed. GA (in weeks): < 28 (*n* = 7), 28–30 (*n* = 11), > 30 (*n* = 10). BW (in grams): < 1000 (*n *= 8), 1000–1200 (*n *= 10), > 1200 (*n *= 10). PMA (in weeks): < 32 (*n *= 6), 32–34 (*n *= 18), > 34 (*n* = 4). Success was calculated as complete regression of disease without need for any other modality of treatment such as anti-vascular endothelial growth factor (anti-VEGF) or pars plana vitrectomy.

**Results:**

The overall success rate was 94.64% (53/56). Two babies who needed additional modality of treatment were < 28 weeks of GA (one eye) and 28–30 weeks (two eyes). One baby (one eye) was < 1000 gm and the other (two eyes) was > 1200 gm, while PMA at which additional treatment was needed was 30 weeks in one baby (one eye) and 33 weeks in the other (two eyes).

**Conclusion:**

In this era of anti-VEGF treatment, even in cases of APROP, LASER should still be considered as a primary modality of treatment, as it is a one-time treatment without the concern of systemic side effects and recurrent/persistent avascular zones.

##  INTRODUCTION

Aggressive posterior retinopathy of prematurity (APROP) is a distinct variant of retinopathy of prematurity (ROP) which does not respect various stages and can rapidly lead to blindness if untreated. According to various studies,^[1,2,3,4]^ the overall incidence of ROP varies from 38% to 51.9% in the Indian subcontinent. Nearly 26.4% of babies needed treatment for one of the stages of ROP^[4]^ almost half of them had APROP.^[4]^

Many previous studies have shown more unfavorable outcomes in APROP (ranging from 15% to 29%) undergoing laser treatment compared with anti-vascular endothelial growth factor (anti-VEGF) injection.^[5,6,7,8]^ Because of these unfavorable outcomes, alternatives in treatment of APROP were explored over the last few years. The first prospective, controlled, randomized trial was performed by Mintz-Hittner^[9]^ showing significantly lower recurrence rate following intravitreal bevacizumab (IVB), compared to laser photocoagulation, especially in Zone 1 ROP. After that, several studies reported the success rate of around 85% in Zone 1 and APROP with IVB monotherapy.^[10,11]^ However, none of these studies talked about the systemic side effects, recurrence rates, and need of laser treatment after IVB in their studies.

In this study, we prospectively studied the structural success rate in cases of APROP after laser treatment and assessed various parameters like systemic side effects, complications, follow-up period and retreatment.

##  METHODS

A prospective case series including 56 eyes of 28 infants with APROP who were treated with laser photocoagulation between January 2015 and June 2018 and were followed-up for 12 months. Various parameters including birth weight (BW), gestational age, postmenstrual age (PMA), neonatal illness risk factors, and oxygenation were studied.

**Figure 1:**
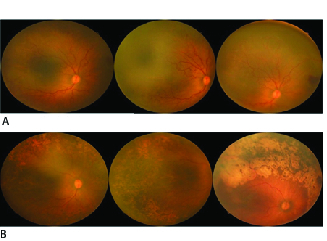
Fundus photos of eyes pre- (A) and post-laser (B).

Diagnosis of APROP was made in accordance with the International Classification of ROP^[12]^ and documented by retinal drawings and/or NEORET images [Figure 1A]. APROP was diagnosed according to the International Classification,^[12]^ which is described as follows: “The characteristic features of this type of ROP are its posterior location, prominence of plus disease, and the ill-defined nature of the retinopathy.”

**Figure 2:**
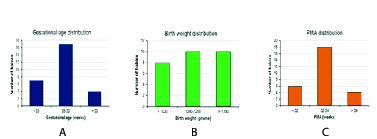
Column chart showing number of babies according to gestational age (A), birth weight (B), and PMA (C).

**Figure 3:**
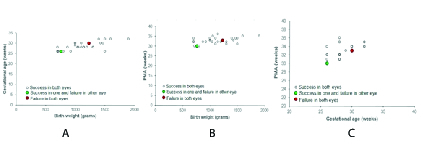
Scatter plot showing success corresponding to birth weight and gestational age (A), birth weight and PMA (B), and gestational age and PMA (C).

**Figure 4:**
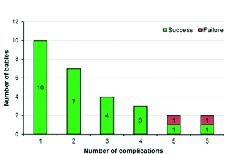
Stacked bar chart showing number of babies according to number of risk factors.

All infants received confluent laser spots (less than half burn width apart) to cover the full avascular retina with green laser (532 nm) delivered through the indirect ophthalmoscopic system under topical anesthesia in the NICU setting. Mean number of laser spots were 4200 +/– 600 per eye. Completion of laser was confirmed by a second observer or NEORET photography. PMA at treatment, number of laser sittings, and outcome were noted. Babies were followed-up for up to 12 months and the outcome was labeled as unfavorable if any of the following three situations/conditions were seen: (1) retinal detachment (stage 4a/4b/5), (2) falciform fold involving the macula, and (3) disc/macular dragging.

### Follow-up and Retreatment

The follow-up examination after laser treatment with NEORET photography [Figure 1B] were performed weekly for one month, biweekly for two months, and then every three months for nine months. In cases where avascular non-covered areas were seen, fill-in laser was applied to the skipped areas usually one or two weeks after the initial laser session. Mean number of laser spots during retreatment was 400 /+– 100 per eye.

### Statistical Methods

The description for various risk factors such as gestational age, BW, PMA, and regression week were obtained in terms of mean and standard deviation. The unfavorable event was treated as dependent variable and the effect of different risk factors was studied on outcome through univariate logistic regression. The odds ratio associated with each factor were obtained and interpreted. The number of babies with different complications were obtained and the association of outcome with the number of complications was represented through a stacked bar chart. Further, the scatter plots showing association of BW with gestational age and PMA were obtained highlighting the unfavorable events. All analyses were performed using SPSS version 20.0 (IBM Corp, USA) and the graphical representations were obtained using Microsoft Excel. A *p*-value of less than 5% was considered as statistically significant.

##  RESULTS

Fifty-six eyes of 28 preterm babies (21 male and 7 female) with APROP who underwent laser treatment were studied. The mean gestational age of mothers was 28.5 (SD: 2.01) weeks and the mean BW of babies was 1,128 (SD: 310) gr. The mean PMA of mothers was 32.7 (SD: 1.63) weeks. The undesired event occurred in three eyes out of 56 resulting in the overall success rate of 94.64%. The risk associated with various factors was studied though logistic regression and showed the following results.

### Gestational age

The odds associated with gestational age was 1.036 (95% CI: 0.577–1.858) indicating hardly any effect of gestational age on the success rate, and the effect was not statistically significant (*P* = 0.906).

### BW

The odds associated with BW was 0.999 (95% CI: 0.995–1.003) indicating hardly any effect of BW on the success rate, and moreover the effect was statistically insignificant (*P* = 0.750)

### PMA

The odds associated with PMA was 0.735 (95% CI: 0.339–1.591) indicating that the increase in PMA reduced the risk of failure; however, the effect was not statistically significant (*P* = 0.434).

### Regression week

It was defined as the interval between application of laser to complete regression of the disease. The odds associated with regression week was 3.814 (95% CI: 0.446–32.641) indicating that any increase in the regression week increased the risk of failure, although the effect was found to be statistically insignificant (*P* = 0.222).

Figures 2A–2C show the distribution of babies according to gestational age, BW, and PMA. With regard to gestational age, the majority, that is, 17 (60.7%) babies were born in 28–30 weeks of gestational age. There were 20 (71.42%) babies with BW > 1000 gm; and there were 18 (64.29%) babies with mothers having PMA between 32 and 34 weeks.

Figures 3A–3C, show the scatter plots demonstrating the structural success rates corresponding to the interplay between GA, PMA, and BW.

Supplementary laser treatment was needed in 20 eyes, one to two weeks after the initial laser treatment. The mean regression period was 4.68 weeks (SD: 0.71) in babies with BW > 1000 gm and 6.36 weeks (SD: 0.92) when the BW was < 1000 gm. No relation was seen with GA or PMA.

Out of the 56 eyes, 53 eyes had complete regression while of the other three eyes, two progressed to stage 5 and one progressed to stage 4A despite laser. One baby (two eyes), which progressed to stage 5 was 30 weeks of GA and > 1200 gm BW, while the other baby (one eye) that progressed to stage 4A was < 28 weeks and < 1000 gm BW.

Table 1 illustrates the distribution of various risk factors/systemic illness in babies. Supplemental unmonitored oxygen was the common factor among all the babies. In Figure 4, the stacked bar chart representation reveals that as the number of risk factors increases, the likelihood of failure increases. Failures were observed in cases with five or more complications.

**Table 1:** Number of babies with different complications


**Complication**	**Number**	**%**
Oxygen administration	28	100
Neonatal jaundice	11	39.28
Sepsis	10	35.71
RDS	11	39.28
Hypothermia	5	17.86
Shock	3	10.71
Blood transfusion	2	7.14

##  DISCUSSION

In the present study, 53 out of 56 eyes had complete regression, while out of 3 eyes without complete regression, 2 progressed to stage 5 and 1 progressed to stage 4A. Mean regression period was 4.68 weeks in babies with BW > 1000 gm and 6.36 weeks when BW was < 1000 gm. According to previous studies, mean GA and BW of infants with APROP were significantly lower than those with non-APROP.^[13,14]^ A study by Tong et al^[15]^ showed that older PMA and low neutrophil count were associated as risk factors for retinal detachment in APROP. Also, low BW was significantly associated with recurrence in APROP. Many studies have shown the beneficial effect of IVB over laser in terms of structural outcome specially in cases of APROP. The studies by Drenser et al^[7]^ and Pandya et al^[8]^ reported that 8/44 eyes and 3/6 eyes, respectively, progressed to retinal detachment in spite of laser treatment. While 17% of eyes landed into retinal detachment in the study by Sanghi et al,^[6]^ in our study only 5.36% of babies progressed to Stage 4/5 after laser treatment which is much less than the rates reported by previous studies.

Gunay et al from Turkey reported 0/25 and 2/11 eyes progressing to retinal detachment in IVB group versus laser group^[10]^ while Nicoara et al reported the success rate of 94% in IVB group compared to 83% in laser group in the Romanian population.^[11]^

Recently, many studies have reported reactivation of disease with IVB. In a study by Lorenz et al,^[16]^ only 25% of eyes with APROP showed regression with lower doses of bevacizumab, that is, 0.312 mg. In a recent study, Mintz-Hittner showed reactivation in 100% of eyes with APROP^[17]^ while Blair et al found 41% reactivation in APROP eyes in their study.^[18]^ This difference in reactivation may be because of higher levels of VEGFs in APROP eyes. Because of late reactivation up to 69 weeks+ after anti-VEGF treatment, longer-term follow-up is required.^[19]^ The babies in our study were followed-up for up to one year of age to look for recurrence. The average number of visits in our study was 7–10 in a year. In study by Simona et al,^[20]^ babies receiving laser needed an average follow-up of up to 60 weeks with an average of 8–9 visits which is much less compared to a follow-up of up to 80 weeks and an average of 16–18 visits in the group receiving anti-VEGF treatment. Persistent avascular zones after IVB have been reported in up to 91.7% of cases by Leopore et al,^[21]^, while 100% of eyes needed additional treatment with laser because of persistent avascular zones which were confirmed on fluorescein angiography in a study by Michael Blair et al.^[18]^ In the present study, 53 eyes after regression did not need any additional form of treatment in the form of anti-VEGF injection or surgery. Another concern is of the “crunch phenomenon” with IVB which causes fibrovascular contraction and tractional retinal detachment following intravitreal anti-VEGF.^[22]^ Systemic safety is another concern. Intravitreal injection causes breakdown of blood retinal barrier,^[23]^ hence, anti-VEGF is found in systemic circulation after intravitreal injection causing serum VEGF levels to decrease.^[23,24]^ Various studies demonstrated that serum VEGF plasma levels may be lowered up to two to seven weeks after IVB.^[25,26,27]^ These decreased VEGF levels may cause adverse effects on VEGF-dependent developments such as the development of brain, lungs, kidney, and normal neural retinal development.^[28]^ Few studies have shown delay in growth and pulmonary dysplasia in bevacizumab-treated babies.^[29]^ A study by Morin et al reported that severe neurodevelopmental disabilities (Bayley scores < 70) was seen 3.1 times higher with bevacizumab compared to laser.^[30]^ Most recently, hypotension-related reports^[31]^ and histopathologically proven new or reactivation of bronchopulmonary dysplasia have been noted after anti-VEGF administration.^[32]^

Few studies were designed to study specific abnormalities related to anti-VEGF treatment and detected no systemic complications.^[29,33,34]^ These negative results may be due to the fact that infants with ROP present more often with developmental disorders compared to other infants, causing difficulty in assessing systemic side effects of anti-VEGF treatment.^[35]^ None of the babies showed any signs of systemic complications in our case series. Laser therapy in ROP is associated with restricted visual field^[36,37,38,39]^ and refractive error.^[40,41]^ It is postulated that increased laser ablation spots might induce more severe myopia and for every 100 laser spots myopia increases by –0.14 ± 0.05D.^[42]^ Prevalence of high myopia reported in literature varies from 8%^[43]^ to 35%.^[44]^ Various studies have shown more chance of refractive anisometropia^[43]^ and myopia^[40,41]^ in babies receiving laser therapy compared to IVB. However, the study by Kua et al^[42]^ and Isaac et al^[44]^ reported no statistically significant difference in refractive error between IVB and laser groups. Because of conflicting results, one meta-analysis^[45]^ has concluded the need to investigate the long-term effects of IVB therapy on refractive error development. Although, myopia^[40,41]^ and field restriction^[36,37,38,39]^ are the main adverse events associated with laser therapy, in our study, we only evaluated the structural success and the refractive status was not evaluated.

This study had a few limitations; it was a non-comparative study with limited follow-up period of one year. Due to being non-comparative, the refractive status was not evaluated and because of the small sample size, no significant association between BW, GA, PMA, and risk factor could be detected.

In summary, approximately 10% of ROP cases in the Indian subcontinent is APROP.^[4]^ Need for long-term follow-up and retreatment, concern about systemic complications and financial constraints still loom large in the Indian subcontinent where compliance is a big challenge. Laser therapy can be considered as primary modality of treatment specially in patients with expected poorer compliance and financial constraints.

##  Financial Support and Sponsorship

Nil.

##  Conflicts of Interest

There are no conflicts of interest.
